# PET imaging of noradrenaline transporters in Parkinson’s disease: focus on scan time

**DOI:** 10.1007/s12149-018-1305-5

**Published:** 2018-10-06

**Authors:** Joachim Brumberg, Johannes Tran-Gia, Constantin Lapa, Ioannis U. Isaias, Samuel Samnick

**Affiliations:** 1 Department of Nuclear Medicine, University Hospital and Julius-Maximilians-University, Oberdürrbacher Straße 6, 97080 Würzburg, Germany; 2 Department of Neurology, University Hospital and Julius-Maximilians-University, Josef-Schneider-Straße 11, 97080 Würzburg, Germany

**Keywords:** Parkinson’s disease, Locus coeruleus, Noradrenaline transporter, Positron emission tomography

## Abstract

**Objective:**

In subjects with idiopathic Parkinson’s disease (PD) the functional state of the locus coeruleus and the subtle derangements in the finely tuned dopamine–noradrenaline interplay are largely unknown. The PET ligand (*S,S*)-[^11^C]-*O*-methylreboxetine (C-11 MRB) has been described to reliably bind noradrenaline transporters but long scanning protocols might hamper its use, especially in patients with PD. We aimed to assess the feasibility of reducing C-11 MRB scans to 30 min.

**Methods:**

Ten patients with idiopathic PD underwent dynamic C-11 MRB PET (120 min duration) and brain magnetic resonance imaging. Model-based (i.e., simplified and multilinear reference tissue model 2) non-displaceable binding potentials (BP) of selected brain regions were analyzed for a 90 min scan protocol and compared with BP derived from static 30-min data with different starting times (30, 40, 50 and 60 min) after C-11 MRB injection. Intraclass correlation coefficient and linear regression analysis were used to explore the association between BP of different scan durations. Spearman’s *ρ* served to describe the correlation of BP with demographic and clinical parameters.

**Results:**

With respect to kinetic models, BP_50–80_ and BP_60–90_ showed the best correlation in several brain areas (*R*^2^ range 0.95–98; *p* < 0.001). The thalamus showed the highest BP on average. No correlation between BP, clinical and demographic characteristics was observed.

**Conclusions:**

An acquisition time of 30 min, starting 50 or 60 min after C-11 MRB injection, allows a reliable estimation of noradrenaline transporter binding values in Parkinsonian people. A short acquisition time can significantly reduce the discomfort of Parkinsonian patients and facilitate PET studies, especially in the medication-off-state.

## Introduction

Parkinson’s disease (PD) has been historically identified as a nigrostriatal dopamine deficiency syndrome. The dramatic response of most motor and some non-motor symptoms to dopaminergic therapies, coupled with milestone achievements in the understanding of the dysfunction of dopamine homeostasis, furthered this belief [1–3]. However, dopamine neurotransmission may not be the first nor the major neurotransmitter casualty in the pathophysiology of PD [4]. In this respect, there is increasing awareness of variable derangements of non-dopaminergic modulatory systems (e.g., noradrenaline, acetylcholine, and glutamate). However, the general belief is still that the symptoms of PD parallel a neurodegenerative process [4, 5], although certain symptoms relate more to adaptive changes and compensatory adjustments than to slowly progressive dopaminergic failure. As an example, levodopa-related dyskinesia might be due to aberrant release of dopamine from serotonin neurons [6], or preserved striatal cholinergic functions [7]. We also advanced the hypothesis that parkinsonian tremor could result from increased noradrenergic activity arising from the locus coeruleus (LC) [8], which could also be responsible for the benign progression of PD in patients with tremor, owing to a putative neuroprotective and compensatory activity of noradrenaline on its target neurons (including the substantia nigra and the striatum) [9, 10]. This hypothesis has recently been supported by a preliminary study in PD patients with positron emission tomography (PET) using (*S,S*)-^11^C-2-(α-(2-methoxyphenoxy)benzyl)morpholine, also called (*S,S*)-[^11^C]-*O*-methylreboxetine (C-11 MRB), a selective ligand for noradrenaline reuptake transporters (NET), which are localized on noradrenergic synaptic terminals [11]. Information on the noradrenergic terminal density in people with PD is largely missing and our knowledge derives from histopathological examinations, mostly performed in advanced stages of the disease, when compensatory mechanisms are eventually exhausted. Molecular imaging studies in vivo are needed, but have long been hampered by the lack of suitable radioligands. One imaging study used the tracer C-11 RTI-32 to specifically address the noradrenergic activity of the central nervous system in PD patients. The authors showed reduced catecholamine transporter density in the LC and in several regions of the limbic system in depressed PD patients [12]. Recently, Nahimi et al. [11] evaluated the suitability of the ligand C-11 MRB for use in PD patients and observed decreased binding in the thalamus and nucleus ruber, particularly in akinetic-rigid dominant PD patients. Furthermore, a widespread reduced binding of C-11 MRB was found in PD patients with REM sleep behavior disorders, which correlated with the amount of REM sleep without atonia [13]. C-11 MRB was developed by Ding et al. [14, 15] for the exploration of NET availability in patients with psychiatric disorders [16–18]. The main practical limitation of C-11 MRB is the long scan duration; the published scanning protocols comprise dynamic PET scans over at least 90 min [11, 17, 18]. Long protocols, however, cause major discomfort for patients with PD, especially when pausing dopaminergic medication. Arterial input function measurements, to quantify the volume of distribution, could be less of a problem by replacing with the simplified reference tissue model (SRTM) 2 [19] or Ichise’s multilinear reference tissue model (MRTM) 2 [20, 21]. These models, as well as the use of the caudate or the occipital cortex as possible reference regions, have recently been validated in people with PD [11]. The aim of this study was to investigate the validity of shorter scanning protocols for C-11 MRB PET in PD patients. We compared non-displaceable binding potentials (BP) of cortical and subcortical brain regions estimated using a 30-min scan duration that started between 30 and 60 min post-injection, with the model-derived binding values obtained by SRTM2 and MRTM2. Furthermore, we tested region-specific BP for correlations with demographic and clinical parameters.

## Materials and methods

### Patients

We consecutively recruited eight men and two women with a diagnosis of PD according to the UK Brain Bank Clinical Diagnostic criteria for PD. Disease severity and stage was assessed with the Unified Parkinson’s Disease Rating Scale (UPDRS) and the Hoehn and Yahr (H/Y) scale. Patients were clinically investigated in the morning after overnight withdrawal (> 12 h) of all dopaminergic drugs (meds-off) and at 1 h (meds-on) after oral intake of 200/50 mg fast-release soluble levodopa/benserazide. Clinical and demographic data are listed in Table 1. Patients with significant comorbidity, previous history of other neurological conditions (e.g., stroke and epilepsy), dementia, depression or sleep disorders (e.g., REM sleep behavior disorder) were not enrolled. None of the patients were taking antipsychotics, antidepressants, or other drugs possibly affecting the noradrenergic system. C-11 MRB was administered on a compassionate use basis in compliance with §37 of the Declaration of Helsinki and The German Medicinal Products Act, AMG §13.2b. MRI was performed as part of the clinical work-up. The study was approved by the local institutional review board (IRB approval: 2017040601). All patients gave written informed consent to the diagnostic procedures.

**Table 1:** Patient characteristics

Patient no.	Sex	Age (years)	DD (years)	H/Y (stage)	UPDRS III meds-off (score)	UPDRS III meds-on (score)	LEDD (mg)
1	M	59	16	2	30	13	405
2	M	59	3	1	14	3	408
3	M	73	2	3	28	10	450
4	M	65	6	3	36	19	720
5	F	54	2	1	11	6	260
6	M	53	4	1	22	15	557
7	F	61	3	1	25	18	450
8	M	53	3	2	20	16	720
9	M	64	2	1	11	8	780
10	M	64	3	1	15	9	310
Mean ± SD		60.5 ± 6.3	4.4 ± 4.2	1.6 ± 0.8	21 ± 8.5	8.3 ± 7.1	50 ± 181

*DD* disease duration, *H/Y* Hoehn and Yahr stage, *UPDRS* Unified Parkinson’s Disease Rating Scale, *LEDD* levodopa-equivalent daily dose

### Preparation of C-11 MRB

(2*S*,3*S*)-Desethylreboxetine used as precursor for radiolabeling and the MRB reference were purchased from ABX (Radeberg, Germany). All solvents and reagents were provided in Ph. Eur. quality from Merck KG (Darmstadt, Germany) or AppliChem GmbH (Darmstadt, Germany).

C-11 MRB was synthesized on a Tracerlab-Fx-C-Pro synthesis unit (GE Medical Systems, Uppsala, Sweden) following the method published previously by Ding et al., with minor modifications [18, 22]. The radiochemical yield was typically between 4.5 and 5.5 GBq per batch with a specific activity, as determined by analytical HPLC and MRB reference, of 40–60 GBq/µmol (EOS) in a total synthesis time of 45 min.

### PET scans

PET scans were obtained with a combined PET/CT scanner (Biograph mCT 64; Siemens Healthineers, Knoxville, TN, USA). Prior to the acquisition, an intravenous catheter was placed in the antecubital vein of the left or right arm. The patients were positioned comfortably in the PET scanner and instructed to remain still for the total duration of the scan. The patient’s head was fixed in a head holder to avoid motion artefacts. After the acquisition of a low-dose CT for attenuation correction (CARE Dose 4D, 80 mAs, 120 kV; matrix: 512 × 512; 2-mm slice thickness; increment: 30 mm/s; rotation time: 0.5 s; pitch index: 0.8), C-11 MRB was administered intravenously as a slow manual bolus injection of 635 ± 29 MBq. Simultaneously with the injection, a dynamic PET acquisition with 120 min duration was initiated using the 3D list mode. After the acquisition, the data were binned in 33 frames of progressively longer durations (6 × 30 s, 3 × 60 s, 2 × 120 s, 22 × 300 s), and PET images were reconstructed iteratively (HD-PET, 24 subsets, 3 iterations; Gaussian filtering: 5.0 mm; matrix: 400 × 400; axial resolution: 2.0 mm; in-plane resolution: 2.04 × 2.04 mm^2^). Acquisition and data reconstruction were performed using dedicated manufacturer software (syngo MI.PET/CT; Siemens Healthineers, Erlangen, Germany).

### MR imaging

Magnetic resonance imaging (MRI) was performed on a 3T whole-body scanner (MAGNETOM Trio; Siemens Healthineers, Erlangen, Germany) equipped with a 12-channel, phased-array head coil for signal reception. In all subjects, a standard cranial MRI protocol was applied, including an isotropic high-resolution structural T1-weighted MP-RAGE-Sequence (Turbo-FLASH 1.0 × 1.0 × 1.0 mm^3^, TR 2530 ms, TE 3.37 ms, FA 9°, TI 1200 ms) for anatomical co-registration with the PET images.

### Image analysis

#### Data preprocessing

All image data were processed and analyzed using PMOD image analysis software version 3.7 (PMOD Technologies Ltd, Zurich, Switzerland). Dynamic PET data were motion-corrected to a late time point image [40–45 min post-injection (p.i.)] of the scan. An average PET image of 20–60 min p.i. of the motion-corrected time series was calculated for each subject. Based on this, the dynamic PET scan was registered and matched to the individuals’ MP-RAGE image volume. Furthermore, four static PET images of 30 min scan time were created for each patient by averaging over six consecutive of these preprocessed frames starting at 30, 40, 50 and 60 min.

#### VOI definition

Three probability maps were obtained, based on the individuals’ MP-RAGE sequence, by applying the segmentation method [23]. Normalization of the MR images was performed via probability maps transformation into the standard anatomical space of the Montreal Neurological Institute (MNI) [24]. Based on the N30R83 maximum probability atlas by Hammers and colleagues [25], 17 target volumes of interest (VOIs) were defined: amygdala (left/right; l/r), brainstem, caudate nucleus (l/r), cerebellum (l/r), frontal lobe (l/r), hippocampus (l/r), parietal lobe (l/r), putamen (l/r) and thalamus (l/r). Two additional atlas-based auxiliary VOIs were created, one containing the regions of the occipital lobe, and a second by merging the left and right thalamus. The transformation between the patient brain and the MNI space was then used to inversely transform the VOIs to the individuals’ MRI and the co-registered PET space. Last, a VOI containing the LC was outlined using previously published atlas coordinates [26].

#### Kinetic modeling

VOI-specific time activity curves (TAC) from 0 to 90 min p.i. were generated from the dynamic PET data for each patient. To determine the efflux rate constant ($k_{2}^{\prime }$), one-parameter MRTM was applied for each of these TACs using the fused thalamus VOI as the transporter-rich region and the occipital lobe as the reference region. Then, the mean $k_{2}^{\prime }$ of all ten patients was calculated and transferred to the two-parameter MRTM2 and SRTM2 (20) to determine binding values (BP_MRTM2_ and BP_SRTM2_) of the eighteen target VOIs for each patient. Additionally, BP of the four static brain PETs was calculated for each of the above regions using average regional uptake values [BP = (mean activity concentration VOI − mean activity concentration occipital cortex)/(mean activity concentration occipital cortex)]. 30-min time frames starting at four different times p.i. were used (BP_30–60_, BP_40–70_, BP_50–80_, BP_60–90_).

### Statistical analysis

Clinical variables and BP estimates were tested for normality using the Shapiro–Wilk test. We calculated (region-wise) the Spearman’s rank correlation coefficient (Spearman’s *ρ*) and the intraclass correlation coefficient (ICC) between BP_30–60_, BP_40–70_, BP_50–80_ or BP_60–90_ and the model-based binding values (BP_MRTM2_ and BP_SRTM2_). A linear regression analysis was used to describe the correlation between binding values provided by the four static 30-min brain PETs and the two model-derived BP_MRTM2_ and BP_SRTM2_ (all regions). The association between the binding value of the thalamus with age and clinical variables [i.e., disease duration, H/Y stage, UPDRS-III score, and levodopa-equivalent daily dose (LEDD)] was evaluated with a multivariate analysis of variance and Spearman’s *ρ*. Of note, we assumed no relevant influence of laterality on time-dependent BP and we pooled left and right hemisphere for all paired brain regions. Significance was set at *p* < 0.05 and corrected for multiple comparisons.

## Results

**Fig. 1:**
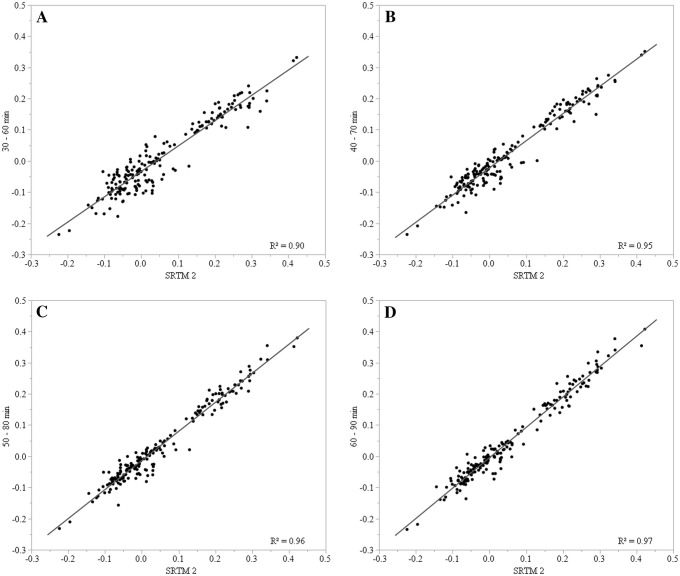
Scatter plots and linear regression analysis of statically derived binding potentials (BP) and binding values estimated with the Simplified Reference Tissue Model 2 (SRTM2). The upper row shows the BP of 30-min scan durations with acquisition start at 30 min (**a**) and 40 min (**b**), the lower row shows BP starting at 50 min (**c**) and 60 min (**d**). All comparisons were plotted on BP estimated with the SRTM2. Regression lines are depicted in grey

**Fig. 2:**
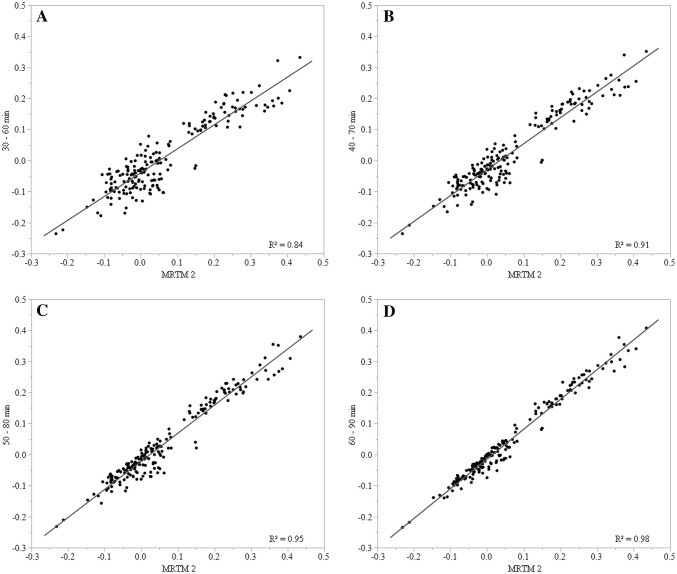
Scatter plots and linear regression analysis of statically derived binding potentials (BP) and binding values estimated with Ichise’s Multilinear Reference Tissue Model 2 (MRTM2). The upper row shows the BP of 30-min scan durations with acquisition start at 30 min (**a**) and 40 min (**b**), the lower row shows BP starting at 50 min (**c**) and 60 min (**d**). All comparisons were plotted on BP estimated with the MRTM2. Regression lines are depicted in grey

Region-wise correlational analyses are listed in Tables 2 and 3. The linear regression analysis is shown in Figs. 1 and 2. With respect to the model-derived binding values, BP_60–90_ (BP_60–90_ = 0.97 × BP_SRTM2_ − 0.01, *R*^2^ = 0.97, *p* < 0.001; BP_60–90_ = 0.96 × BP_MRTM2_ − 0.01, *R*^2^ = 0.96, *p* < 0.00) and BP_50–80_ (BP_50–80_ = 0.93 × BP_SRTM2_ − 0.01, *R*^2^ = 0.96, *p* < 0.001; BP_50–80_ = 0.90 × BP_MRTM2_ − 0.02, *R*^2^ = 0.95, *p* < 0.001) showed a very high correlation. Less significant prediction values were observed for BP_40–70_ (BP_40–70_ = 0.87 × BP_SRTM2_ − 0.02, *R*^2^ = 0.95, *p* < 0.001; BP_40–70_ = 0.83 × BP_MRTM2_ − 0.03, *R*^2^ = 0.91, *p* < 0.001) and BP_30–60_ (BP_30–60_ = 0.81 × BP_SRTM2_ − 0.03, *R*^2^ = 0.90, *p* < 0.001; BP_30–60_ = 0.77 × BP_MRTM2_ − 0.04, *R*^2^ = 0.84, *p* < 0.001). The highest average BP value was found in the thalamus, followed by the putamen and the LC. The average thalamic NET binding potential ranged between 0.22 and 0.27 for BP_MRTM2_, BP_SRTM2_, BP_50–80_ and BP_60–90_, whereas it was lower for BP_30–60_ and BP_40–70_ (0.17 and 0.19). No significant correlations between BP, demographic, and clinical parameters were found.

**Table 2:** Correlations between regional BP estimated with the Simplified Reference Tissue Model 2 (SRTM2) and BP of 30-min scan durations

Region	BP_SRTM2_	BP_30–60_			BP_40–70_			BP_50–80_			BP_60–90_		
	Mean ± SD	Mean ± SD	*ρ*	ICC	Mean ± SD	*ρ*	ICC	Mean ± SD	*ρ*	ICC	Mean ± SD	*ρ*	ICC
Amygdala	− 0.01 ± 0.07	− 0.09 ± 0.04	0.65^#^	0.55	− 0.07 ± 0.04	0.78*	0.72	− 0.05 ± 0.05	0.86*	0.82	− 0.03 ± 0.06	0.92*	0.95
Brainstem	− 0.07 ± 0.03	− 0.11 ± 0.03	0.62	0.54	− 0.09 ± 0.03	0.87°	0.75	− 0.07 ± 0.03	0.95*	0.93	− 0.06 ± 0.03	0.95*	0.91
Caudate	− 0.03 ± 0.08	− 0.10 ± 0.06	0.90*	0.74	− 0.08 ± 0.06	0.90*	0.84	− 0.07 ± 0.07	0.91*	0.90	− 0.06 ± 0.07	0.89*	0.92
Cerebellum	− 0.04 ± 0.04	− 0.01 ± 0.04	0.95*	0.90	− 0.03 ± 0.04	0.97*	0.97	− 0.04 ± 0.04	0.97*	0.99	− 0.04 ± 0.04	0.94*	0.98
Frontal lobe	0.02 ± 0.04	0.00 ± 0.03	0.92*	0.83	0.00 ± 0.03	0.95*	0.91	0.01 ± 0.03	0.98*	0.95	0.01 ± 0.04	0.89*	0.96
Hippocampus	− 0.04 ± 0.04	− 0.06 ± 0.03	0.49^#^	0.67	− 0.04 ± 0.03	0.54^#^	0.78	− 0.03 ± 0.03	0.61°	0.85	− 0.02 ± 0.04	0.78*	0.85
Locus coeruleus	0.19 ± 0.07	0.12 ± 0.04	0.84*	0.55	0.15 ± 0.06	0.84*	0.83	0.18 ± 0.07	0.94*	0.96	0.20 ± 0.07	0.91*	0.98
Parietal lobe	− 0.04 ± 0.04	− 0.06 ± 0.03	0.90*	0.90	− 0.05 ± 0.04	0.94*	0.95	− 0.05 ± 0.04	0.96*	0.98	− 0.04 ± 0.04	0.97*	0.99
Putamen	0.25 ± 0.07	0.18 ± 0.06	0.88*	0.77	0.19 ± 0.06	0.91*	0.85	0.21 ± 0.07	0.89*	0.92	0.22 ± 0.07	0.93*	0.94
Thalamus	0.24 ± 0.06	0.17 ± 0.04	0.84*	0.54	0.19 ± 0.04	0.91*	0.78	0.22 ± 0.05	0.93*	0.94	0.24 ± 0.06	0.94*	0.98

BP of each starting time (30, 40, 50 and 60 min) is compared to BP estimated with SRTM2

*BP* binding potential, *ρ* Spearman’s rank correlation coefficient, *ICC* intra class correlation coefficient, *SD* standard deviation

^#^*p* < 0.05, °*p* < 0.01, *p < 0.001

**Table 3:** Correlations between regional BP estimated with Ichise’s Multilinear Reference Tissue Model 2 (MRTM2) and BP of 30-min scan durations

Region	BP_MRTM2_	BP_30–60_			BP_40–70_			BP_50–80_			BP_60–90_		
	Mean ± SD	Mean ± SD	*ρ*	ICC	Mean ± SD	*ρ*	ICC	Mean ± SD	*ρ*	ICC	Mean ± SD	*ρ*	ICC
Amygdala	0.03 ± 0.07	− 0.09 ± 0.04	0.46^#^	0.31	− 0.07 ± 0.04	0.65°	0.46	− 0.05 ± 0.05	0.79*	0.59	− 0.03 ± 0.06	0.94*	0.83
Brainstem	− 0.03 ± 0.03	− 0.11 ± 0.03	0.31	0.17	− 0.09 ± 0.03	0.65^#^	0.32	− 0.07 ± 0.03	0.83°	0.58	− 0.06 ± 0.03	0.88*	0.82
Caudate	− 0.04 ± 0.08	− 0.10 ± 0.06	0.87*	0.79	− 0.08 ± 0.06	0.93*	0.90	− 0.07 ± 0.07	0.99*	0.96	− 0.06 ± 0.07	0.98*	0.98
Cerebellum	− 0.04 ± 0.04	− 0.01 ± 0.04	0.87*	0.83	− 0.03 ± 0.04	0.92*	0.93	− 0.04 ± 0.04	0.97*	0.98	− 0.04 ± 0.04	0.99*	1.00
Frontal lobe	0.02 ± 0.04	0.00 ± 0.03	0.73*	0.81	0.00 ± 0.03	0.83*	0.90	0.01 ± 0.03	0.93*	0.96	0.01 ± 0.04	0.98*	0.99
Hippocampus	0.00 ± 0.04	− 0.06 ± 0.03	0.42	0.29	− 0.04 ± 0.03	0.52^#^	0.42	− 0.03 ± 0.03	0.65°	0.67	− 0.02 ± 0.04	0.90*	0.88
Locus coeruleus	0.20 ± 0.08	0.12 ± 0.04	0.84*	0.54	0.15 ± 0.06	0.80*	0.80	0.18 ± 0.07	0.94*	0.96	0.20 ± 0.07	0.94*	0.99
Parietal lobe	− 0.04 ± 0.04	− 0.06 ± 0.03	0.89*	0.89	− 0.05 ± 0.04	0.95*	0.95	− 0.05 ± 0.04	0.96*	0.98	− 0.04 ± 0.04	0.99*	0.99
Putamen	0.23 ± 0.08	0.18 ± 0.06	0.87*	0.80	0.19 ± 0.06	0.94*	0.88	0.21 ± 0.07	0.97*	0.95	0.22 ± 0.07	0.97*	0.98
Thalamus	0.27 ± 0.08	0.17 ± 0.04	0.78*	0.37	0.19 ± 0.04	0.88*	0.57	0.22 ± 0.05	0.91*	0.77	0.24 ± 0.06	0.96*	0.90

BP of each starting time (30, 40, 50 and 60 min) is compared to BP estimated with MRTM2

*BP* binding potential, *ρ* Spearman’s rank correlation coefficient, *ICC* intra class correlation coefficient, *SD* standard deviation

^#^*p* < 0.05, °*p* < 0.01, *p < 0.001

## Discussion

This study suggests that an acquisition time of 30 min for C-11 MRB PET can offer a reliable estimation of cerebral NET density. This information is of relevance to reduce the scan duration and therefore the discomfort of Parkinsonian patients.

Acquisitions starting at 50 and 60 min showed higher correlation values regarding BP_SRTM2_ and BP_MRTM2_ than the scans starting at 30 and 40 min p.i. (Figs. 1, 2). This finding suggests that for C-11 MRB, many cortical and subcortical regions reach a stable equilibrium at later time points of the scan. An early start combined with a short acquisition duration might partially capture the tracer distribution before the equilibrium has been reached. Additionally, it might not even cover the phase of stable equilibrium occurring at later time points. Accordingly, scans of 30 min duration starting at a later time point (50 or 60 min) showed better ICC values for all tested brain regions (Tables 2, 3). These results are in line with previous PET studies on the noradrenergic system showing stable TACs for the thalamus, the basal ganglia, the cerebellum, and cortical regions between 50 and 90 min p.i. using C-11 MRB [27], C-11 MENET [28], or F-18 FMeNER-D_2_ [29].

Previous studies in non-human primates and humans tested the caudate nucleus, the putamen, and the occipital cortex as potential reference regions for C-11 MRB [30, 31]. In PD patients, only the caudate and the occipital cortex have been validated for this ligand, with the latter showing slightly lower BP [11]. Besides methodological issues [21], we chose the occipital cortex as a reference because of the important role of the caudate nucleus in the pathophysiology of PD. Motor PD symptoms appear when the loss of putamen dopamine level reaches 70–80%, which corresponds to a 50–60% decrease in dopamine neurons [32, 33]. The caudate nucleus is instead relatively spared at early stages of PD [34] and represents a possible site of still-active compensatory and neuroprotective mechanisms upon motor symptoms onset [7], also involving the noradrenergic system arising from the LC [35].

The parameter $k_{2}^{\prime }$ is the clearance rate constant from the reference region to plasma, and needs to be defined a priori for the reference methods SRTM2 and MRTM2 [20]. This parameter can be estimated for each subject by applying the three-parameter model MRTM to the individual TAC of a high-binding region and a reference region (in our case, the thalamus and the occipital brain area, respectively). In the cohort of this study, an average $k_{2}^{\prime }$ of 0.0322 ± 0.0105 min^− 1^ was determined. It is in the range of previously measured values of $k_{2}^{\prime }$ based on one-tissue compartment modeling with arterial input measurements in PD patients and healthy controls [11].

Finally, with regard to the actual NET measurements, the BP_50–80_ and BP_60–90_ shown in this study (Tables 2, 3) are in line with previous reports [11, 13, 18, 28, 29]. Multivariate analysis of variance showed no significant correlation between any BP of noradrenaline transporters and demographic or clinical measures (i.e., disease duration, disease severity and LEDD) in all brain regions investigated. These findings are in line with studies showing a negative correlation between age and BP of the thalamus and LC only in young healthy subjects (mean age: 35 years) [18], but not in the elderly (mean age: 66 years) and PD patients [11]. The small sample size of our study population, as well as the results of previous studies, however, prevent any firm conclusion on demographic and clinical correlations of C-11 MRB binding values. In our case, while disease severity and LEDD were reasonably well represented in our study cohort (Table 1), all but two subjects had disease duration below 5 years and only two females were included in this study. Furthermore, no optical tracking system for motion correction was available, which can considerably improve the analysis of small brain areas such as the LC. Nevertheless, also in agreement with Nahimi et al. [11], it is of relevance to note that C-11 MRB binding was not influenced by the dosage of dopaminergic drugs (i.e., LEDD). This result also parallels previous findings on dopamine reuptake transporter binding values measured with I-123 FP-CIT and SPECT [36]. Further studies are warranted to deepen our understanding of the noradrenergic system in people with PD, with emphasis on clinical phenotypes (e.g., tremor-dominant PD) [37], compensatory and protective mechanisms [8–10].
